# Secondary intraocular lens implantation: a large retrospective analysis

**DOI:** 10.1007/s00417-018-4178-3

**Published:** 2018-11-09

**Authors:** Efstathios Vounotrypidis, Iris Schuster, Marc J. Mackert, Daniel Kook, Siegfried Priglinger, Armin Wolf

**Affiliations:** Department of Ophthalmology, Ludwig-Maximilians-University Munich, Mathildenstrasse 8, 80336 Munich, Germany

**Keywords:** Secondary IOL implantation, Risk factors, Complications, Refractive outcome, Visual outcome

## Abstract

**Purpose:**

To investigate preoperative ocular risk factors and indications for secondary intraocular lens (IOL) implantation and compare postoperative complications, visual and refractive outcomes in a tertiary referral center.

**Methods:**

Patients older than 14 years that underwent secondary IOL implantation and had a minimum follow-up of 3 months were enrolled in this retrospective case series. Preoperative ocular risk factors, indications for surgery, postoperative complications, and visual and refractive outcomes including prediction error (PE) and absolute error (AE) were evaluated. IOLs were fixated in following positions: anterior chamber (AC), retropupillary iris-claw (IC), sulcus, and capsular bag or sclera.

**Results:**

One-hundred eighty-two eyes of 174 patients with mean follow-up of 17 ± 13.6 months were evaluated. Leading cause for surgery was IOL dislocation (75%), followed by secondary aphakia (19%) and IOL opacifications (6%). Previous vitrectomy was the major preoperative ocular risk factor (43%). Mean corrected distance visual acuity improved from preoperative 0.68 ± 0.55 to 0.42 ± 0.31LogMAR by the last follow-up (*p* = 0.001). PE and AE differed highly depending on the indication for surgery (*p* = 0.041 and *p* = 0.008, respectively) and the IOL fixation (*p* = 0.011 and *p* = 0.028, respectively), with IC-IOLs showing the lowest PE and AE. Postoperative AC-hemorrhage occurred mainly after IC-IOLs (*p* = 0.003), and postoperative hypotony was significantly higher in eyes with previous uveitis (*p* = 0.026).

**Conclusions:**

Previous vitrectomy seems to be a major underreported risk factor in eyes that undergo secondary IOL implantation. Refractive outcomes depend on indication for surgery and fixation type, with retropupillary IC-IOLs providing the best refractive results, though not statistically significant compared to other IOL positions.

## Introduction

Ideally, after uneventful cataract surgery, a posterior chamber IOL (PC-IOL) is implanted in the capsular bag. However, this is not always possible, as capsular bag-associated complications may already exist preoperatively (loose zonula, IOL luxation) or occur intraoperatively (anterior or posterior capsular tear). In these cases, either no IOL will be implanted (aphakia) or the IOL has to be fixated in other positions such as anterior chamber (AC), iris, sulcus, or the sclera.

In cases of secondary aphakia or IOL-related complications, a secondary intraocular lens implantation is the preferable surgical procedure. IOL luxation, incorrect IOL power, IOL opacification, uveitis-glaucoma-hyphema (UGH) syndrome, patient dissatisfaction, or secondary aphakia indicate the major reasons for such surgery. Secondary IOL implantations have increased over recent years, and this surgical procedure is now considered common [1, 2].

This retrospective longitudinal case series was conducted in order to identify preoperative ocular risk factors and indications for secondary IOL surgery in a tertiary vitreoretinal referral center and compare the postoperative complications, refractive and visual outcome of such surgery. A secondary analysis examined the influence of preoperative ocular risk factors, biometry, surgeon, IOL fixation, and postoperative complications on the refractive and visual outcome. All secondary intraocular lenses were placed in one of the following positions: anterior chamber (AC, angle supported), retropupillary iris-claw fixation (IC-IOL), sulcus without optic capture, capsular bag, or trans-scleral fixation.

## Material and methods

### Study design

This retrospective analysis reviewed all surgical case logs from 2009 to 2013 at the Department of Ophthalmology, Ludwig-Maximilians-University, Munich (vitreoretinal tertiary referral center) that received a secondary IOL implantation. In order to have a longer adequate follow-up and to avoid different implanted IOLs and new IOL technologies, the study period was limited to 5 years. Patients older than 14 years and with a minimum follow-up of at least 3 months were included in this study. The study was approved by the Institutional Review Board of the Department of Ophthalmology, Ludwig-Maximilian-University, Munich and adhered to the tenets of the Declaration of Helsinki.

### Patients

Every case was reviewed individually. Preoperative data including age, sex, surgery date, laterality, date of primary cataract surgery, indication for secondary IOL implantation; further ocular pathologies like pseudoexfoliation (PXF), uveitis, trauma, systemic syndromes, complete previous vitrectomy or other vitreoretinal surgery (scleral buckling, retinal cryocoagulation); and biometric data, uncorrected and corrected distance visual acuity (UDVA, CDVA), objective refraction, and refractive power of the previously used glasses were collected. Intraoperative parameters included surgeon, position and type of the main incision, capsular bag status, type and power of secondary IOL, fixations position, and anterior vitrectomy. Postoperative UDVA, CDVA, objective, and manifest refraction, as well as the length of the follow up, were documented. Postoperative complications that were noted over the first month were defined as short-termed and after 3 months as long-termed.

### Prediction and absolute error

As defined previously [3], refractive prediction error (PE) was calculated as the difference between postoperative objective refraction expressed as spherical equivalent (ORSE) and the predicted spherical equivalent of the refraction obtained from the preoperative biometry (IOL Master 500, Carl Zeiss Meditec AG, Jena, Germany). Absolute error (AE) was calculated in the standard way as the absolute value of the refractive PE. In this study, the Haigis formula was used for normal and long eyes (≥ 22 mm), the Hoffer-Q for short eyes (< 22 mm), and the SRK-T for pseudophakic and aphakic eyes. Refractive prediction error (PE) and mean absolute error (AE) as well as their standard deviations (SDs), median absolute error (MedAE), and percentages of the eyes within ± 0.5D, ± 1D, and ± 2D of the predicted postoperative refraction are reported in the outcomes of the study.

### Statistical analysis

Tests for data without normal distribution were performed. Non-parametric tests (Mann-Whitney *U*, Wilcoxon rang-sum test) were performed to assess the significance of the differences between pre- and postoperative examinations and non-parametric analysis of variance (Kruskal-Wallis test) for the assessment of differences between multiple grouping variables. The chi-square test was performed as an independency test between categorical data. Groups with less than 10 patients were excluded from the statistical analysis. Refixated IOLs were also excluded from the statistical analysis of visual and refractive outcome. In all cases, the same level of significance was defined (*p* < 0.05). SPSS statistics software package version 23 for Windows (IBM, Armonk, NY, USA) was used for the statistical analysis, and Microsoft Excel (Microsoft Corporation, Redmond, WA, USA) was used for compiling some of the statistical graphs.

## Results

Five-hundred seventy-five eyes underwent a secondary IOL implantation over the examined period of time (2009–2014). However, only a total of 182 eyes (98 right, 84 left) of 174 patients (100 male, 74 female) with a mean age of 63 years old (range 15–92 years old) were evaluated and analyzed in this study, due to lack of follow-up of other patients. Eight patients underwent surgery on both eyes.

The most often reported preoperative risk factor was previous complete pars plana vitrectomy (78 eyes, 43%). Eight of these eyes had, in addition, previous retinal surgery. Overall, previous retinal surgery was noted as the second most frequent preoperative ocular risk factor (62 eyes, 34%), followed by trauma (30 eyes, 17%), PXF (22 eyes, 12%), and uveitis (16 eyes, 9%). Myopia was observed in more than 35% of the cases. Moderate myopia, with an axial length (AL) between 28 and 30 mm, was observed in 10 cases, and high myopia, with an AL > 30 mm, in 7 cases.

**Fig. 1:**
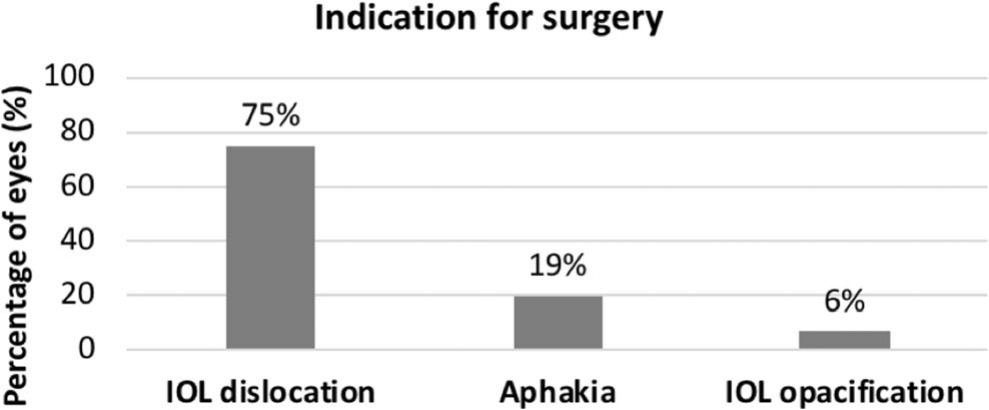
Percentage of eyes for each specific indication for secondary intraocular lens implantation

Figure 1 demonstrates the frequency of each indication for secondary IOL implantation. Leading cause for surgery was IOL dislocation. Specifically, an in-the-bag IOL dislocation was observed in 112 of 134 eyes (84%) and an out-of-the bag IOL dislocation in 22 eyes (16%). Moreover, 47 of 134 (35%) eyes had an IOL dislocation beneath the optical zone, resulting to a high ammetropia of the eye preoperatively.

The mean follow-up time was 17 ± 13.6 months (range 3–60 months). The mean time period between the primary surgery and the secondary IOL implantation was 8.4 ± 6.5 years (range 0–32 years).

Four experienced surgeons performed all the surgical procedures. Surgeon 1 performed 39%, surgeon 2 23%, surgeon 3 20%, and surgeon 4 18% of the surgeries. A scleral incision of different lengths was always performed at the superior part of the eye (position between 70°-120°). During the observation time (2009-2013), the most frequent final position of the implanted IOL was the anterior chamber (angle supported AC-IOL, 92 eyes, 50% of all cases). A retropupillary fixation of an iris-claw IOL (IC-IOL, Verisyse®) was performed in 22% of the cases (40 eyes). A sulcus fixation was available in 15% of the cases (28 eyes), and an in-the-bag fixation was performed in 6% of the cases (10 eyes). A scleral fixation was performed in 7% of the cases (12 eyes). For the IOL power calculation, the biometric formula SRK-T was used in 67% of the cases, followed by the Haigis in 17% and the Hoffer Q in 7%. No biometric formula was used in 9% of the cases, due to re-fixation of the primary implanted IOL.

### Refractive and visual outcome

Eyes, in which a re-fixation of the primary implanted IOL was performed, were excluded from the statistical analysis of this study. Therefore, 165 remaining eyes were evaluated with regard to their visual and refractive outcome. Due to that, only 6 out of 12 scleral fixated IOLs could be evaluated.

Overall, preoperative and postoperative objective spherical equivalent, CDVA, PE, AE with their standard deviation, and median AE with regard to the type of IOL fixation are demonstrated in Table 1. Because of the high standard deviation of the preoperative values, a further classification in aphakic and pseudophakic eyes with regard to the preoperative IOL status was performed, as shown in Tables 2 and 3. Eyes with a dislocation of the IOL optic beneath the optical zone were preoperatively classified as aphakic, whereas eyes with a dislocation of the IOL optic within the optical zone as pseudophakic.

**Table 1:** Preoperative and postoperative corrected distance visual acuity (CDVA), objective refraction spherical equivalent (ORSE), prediction error (PE), and mean absolute error (AE) with regard to the fixation type of the intraocular lens (IOL) in all evaluated eyes. *SD* standard deviation, *CI* confidence interval

Parameter mean ± SD (95% CI)	IOL fixation					
	Anterior chamber (*n* = 85)	Iris (*n* = 39)	Sulcus (*n* = 25)	Capsular bag (*n* = 10)	Sclera (*n* = 6)	All eyes (*n* = 165)
CDVA pre (LogMAR)	0.67 ± 0.43 (0.58–0.77)	0.79 ± 0.74 (0.55–1.03)	0.50 ± 0.53 (0.29–0.72)	0.95 ± 0.55 (0.55–1.35)	0.27 ± 0.23 (0.02–0.52)	0.68 ± 0.55 (0.59–0.76)
CDVA post	0.45 ± 0.23 (0.40–0.50)	0.42 ± 0.48 (0.27–0.58)	0.34 ± 0.26 (0.24–0.45)	0.42 ± 0.23 (0.25–0.59)	0.28 ± 0.16 (0.12–0.45)	0.42 ± 0.31 (0.37–0.47)
Preoperative ORSE (D)	+ 3.17 ± 6.73 (1.57–4.78)	+ 5.74 ± 6.48 (3.36–8.11)	+ 2.93 ± 7.09 (− 0.13 to 6.00)	+ 6.00 ± 7.75 (0.04–11.96)	+ 11.44 ± 2.26 (9.07–13.82)	+ 4.25 ± 6.87 (3.01–5.40)
Postoperative ORSE (D)	− 0.62 ± 1.58 (− 0.96 to − 0.28)	− 0.32 ± 1.32 (− 0.75 to 0.11)	− 1.11 ± 1.35 (− 1.66 to − 0.55)	− 0.96 ± 1.94 (− 2.35 to 0.42)	− 0.06 ± 0.83 (− 0.81 to 0.94)	− 0.62 ± 1.50 (− 0.85 to − 0.39)
PE (D)	− 0.01 ± 1.43 (− 0.31 to 0.30)	− 0.11 ± 1.06 (− 0.45 to 0.24)	+0.77 ± 1.46 (0.16–1.37)	+0.71 ± 1.68 (− 0.49 to 1.91)	+0.14 ± 0.75 (− 0.65 to 0.93)	+ 0.14 ± 1.38 (− 0.08 to 0.35)
AE (D)	+ 1.02 ± 0.99 (0.80–1.23)	+ 0.81 ± 0.68 (0.59–1.03)	+ 1.37 ± 0.89 (1.00–1.73)	+ 1.42 ± 1.07 (0.65–2.19)	+ 0.62 ± 0.37 (0.23–1.00)	+ 1.03 ± 0.92 (0.89–1.17)

**Table 2:** Preoperative and postoperative corrected distance visual acuity (CDVA), objective refraction spherical equivalent (ORSE), prediction error (PE), mean absolute error (AE) and median absolute error (MedAE) with regard to the fixation type of the intraocular lens (IOL) in all eyes that were preoperatively considered as aphakic. *SD* standard deviation, *CI* confidence interval

Parameter mean ± SD (95% CI)	IOL fixation					
	Anterior chamber (*n* = 37)	Iris (*n* = 18)	Sulcus (*n* = 6)	Capsular bag (*n* = 6)	Sclera (*n* = 6)	All eyes (*n* = 73)
CDVA pre (LogMAR)	0.73 ± 0.41 (0.60–0.87)	0.93 ± 0.84 (0.52–1.35)	0.90 ± 0.70 (0.16–1.64)	0.92 ± 0.59 (0.29–1.54)	0.27 ± 0.23 (0.02–0.51)	0.77 ± 0.59 (0.64–0.91)
CDVA post (LogMAR)	0.47 ± 0.22 (0.40–0.54)	0.34 ± 0.18 (0.26–0.43)	0.45 ± 0.29 (0.15–0.75)	0.35 ± 0.24 (0.10–0.60)	0.28 ± 0.16 (0.12–0.45)	0.41 ± 0.22 (0.36–0.46)
Preoperative ORSE (D)	+ 7.99 ± 7.47 (5.20–10.78)	+ 11.30 ± 3.33 (9.53–13.08)	+ 14.22 ± 6.46 (6.21–22.24)	+ 11.9 ± 4.52 (6.29–17.51)	+ 11.44 ± 2.26 (9.07–13.82)	+ 10.00 ± 6.18 (8.43–11.57)
Postoperative ORSE (D)	− 0.97 ± 1.87 (− 1.59 to − 0.34)	− 0.24 ± 1.29 (− 0.89 to − 0.40)	− 0.69 ± 1.78 (− 2.56–1.19)	− 0.04 ± 1.80 (− 1.93–1.85)	0.06 ± 0.83 (− 0.81–0.94)	− 0.60 ± 1.67 (− 0.99 to − 0.21)
PE (D)	+ 0.27 ± 1.69 (− 0.29–0.83)	− 0.09 ± 1.18 (− 0.68–0.49)	+ 0.63 ± 1.98 (− 1.44–2.71)	+ 0.03 ± 1.67 (− 1.72–1.78)	+ 0.14 ± 0.75 (− 0.65–0.93)	+ 0.18 ± 1.52 (− 0.18–0.53)
MAE (D)	+ 1.18 ± 1.22 (0.77–1.59)	+ 0.91 ± 0.73 (0.55–1.27)	+ 1.75 ± 0.85 (0.85–1.82)	+ 1.21 ± 1.01 (0.15–2.28)	+ 0.62 ± 0.37 (0.23–1.00)	+ 1.12 ± 1.03 (0.88–1.36)

**Table 3:** Preoperative and postoperative corrected distance visual acuity (CDVA), objective refraction spherical equivalent (ORSE), prediction error (PE), mean absolute error (AE) and median absolute error (MedAE) with regard to the fixation type of the intraocular lens (IOL) in all eyes that were preoperatively considered as pseudophakic. *SD* standard deviation, *CI* confidence interval

Parameter mean ± SD (95% CI)	IOL fixation				
	Anterior chamber (*n* = 48)	Iris (*n* = 21)	Sulcus (*n* = 19)	Capsular bag (n = 4)	All eyes (*n* = 92)
CDVA pre (LogMAR)	0.62 ± 0.45 (0.49–0.75)	0.67 ± 0.65 (0.37–0.96)	0.38 ± 0.40 (0.18–0.57)	1.00 ± 0.57 (0.09–1.91)	0.60 ± 0.51 (0.49–0.70)
CDVA post (LogMAR)	0.43 ± 0.24 (0.37–0.50)	0.48 ± 0.63 (0.20–0.77)	0.32 ± 0.24 (0.19–0.43)	0.53 ± 0.21 (0.20–0.85)	0.42 ± 0.36 (0.35–0.50)
Preoperative ORSE (D)	− 0.44 ± 2.70 (− 1.30–0.42)	− 0.20 ± 2.21 (− 1.43–1.03)	− 0.20 ± 2.69 (− 1.54–1.14)	− 1.38 ± 1.53 (− 3.82–1.07)	− 0.39 ± 2.53 (− 0.96–0.19)
Postoperative ORSE (D)	− 0.36 ± 1.28 (− 0.73–0.15)	− 0.39 ± 1.37 (− 1.01–0.24)	− 1.24 ± 1.21 (− 1.82 to − 0.65)	− 2.34 ± 1.27 (− 4.36 to − 0.33)	− 0.63 ± 1.36 (− 0.91 to − 0.35)
PE (D)	− 0.22 ± 1.17 (− 0.55–0.12)	− 0.12 ± 0.98 (− 0.57–0.33)	+0.81 ± 1.32 (0.17–1.44)	1.73 ± 1.23 (− 0.24–3.69)	+0.10 ± 1.26 (− 0.16–0.36)
MAE (D)	+ 0.89 ± 0.78 (0.66–1.12)	+ 0.73 ± 0.65 (0.43–1.03)	+ 1.25 ± 0.88 (0.82–1.67)	1.73 ± 1.23 (− 0.24–3.69)	+ 0.96 ± 0.82 (0.79–1.13)

**Fig. 2:**
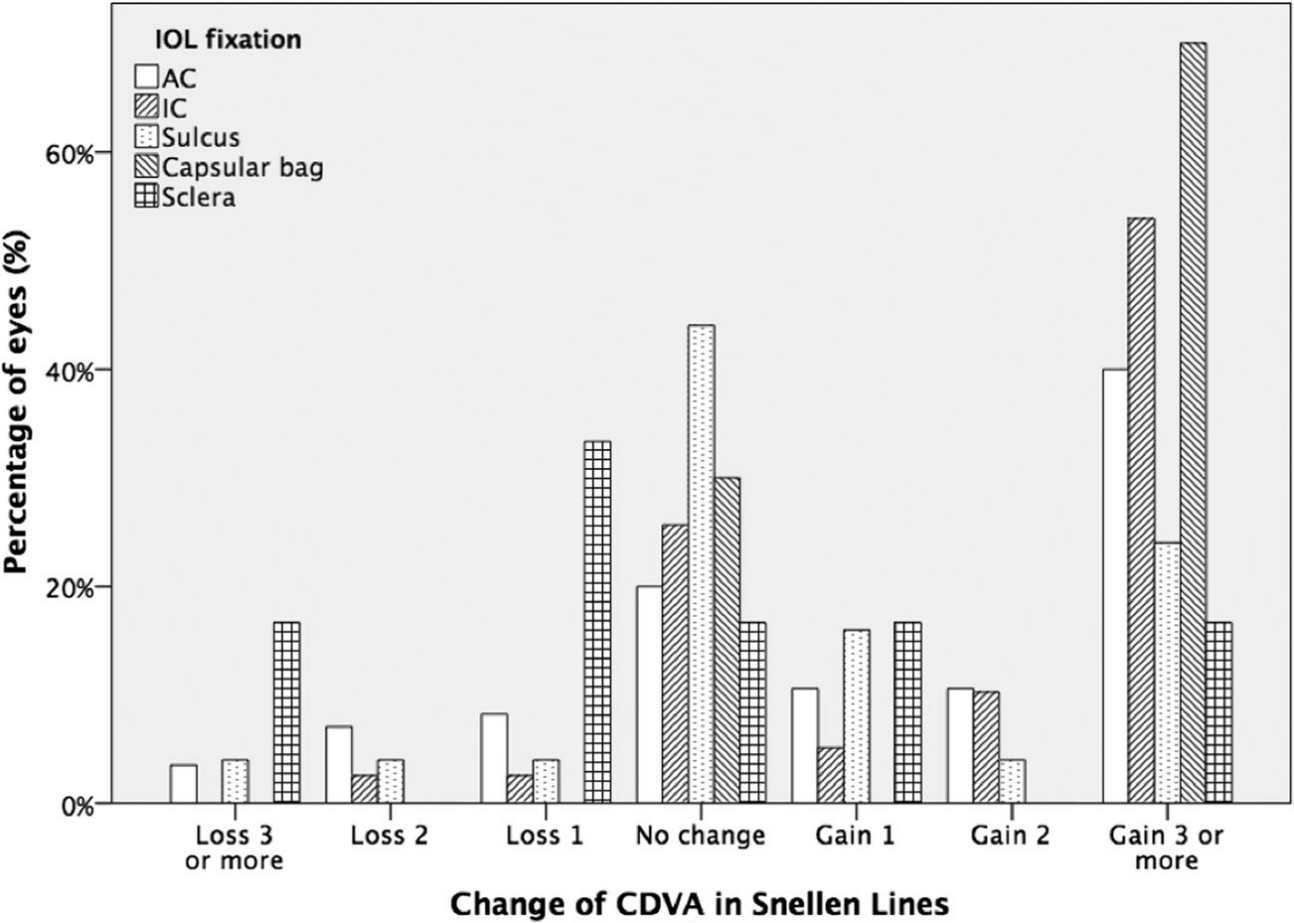
Percentage of eyes with change in their corrected distance visual acuity (CDVA) in Snellen lines with regard to the type of IOL fixation. AC anterior chamber, IC iris-claw

Preoperatively, the trans-scleral fixated IOLs showed the better CDVA than the other groups, whereas the worst CDVA was noted in the capsular-bag implanted IOLs (*p* = 0.009, Kruskal-Wallis) as shown in Table 1. Mean CDVA of all eyes improved from preoperative 0.68 ± 0.55 LogMAR to 0.42 ± 0.31 by the last follow-up (*p* < 0.001, Wilcoxon test), resulting in no statistically significant difference between the groups postoperatively (*p* = 0.062, Kruskal-Wallis). The majority of the eyes (99 eyes, 60%) showed an improvement in visual acuity (gain of one line or more), 42 remained stable (26%) and 24 showed a postoperative visual loss of one line or more (15%). CDVA improved by a statistically significant degree in every group except for the sclera fixated IOLs (*p* = 0.098, Wilcoxon Test), where preoperative CDVA was already quite good, as shown in Table 1. Figure 2 displays the percentage of the eyes according to the change in CDVA with regard to the IOL fixation.

### Prediction and absolute error

**Fig. 3:**
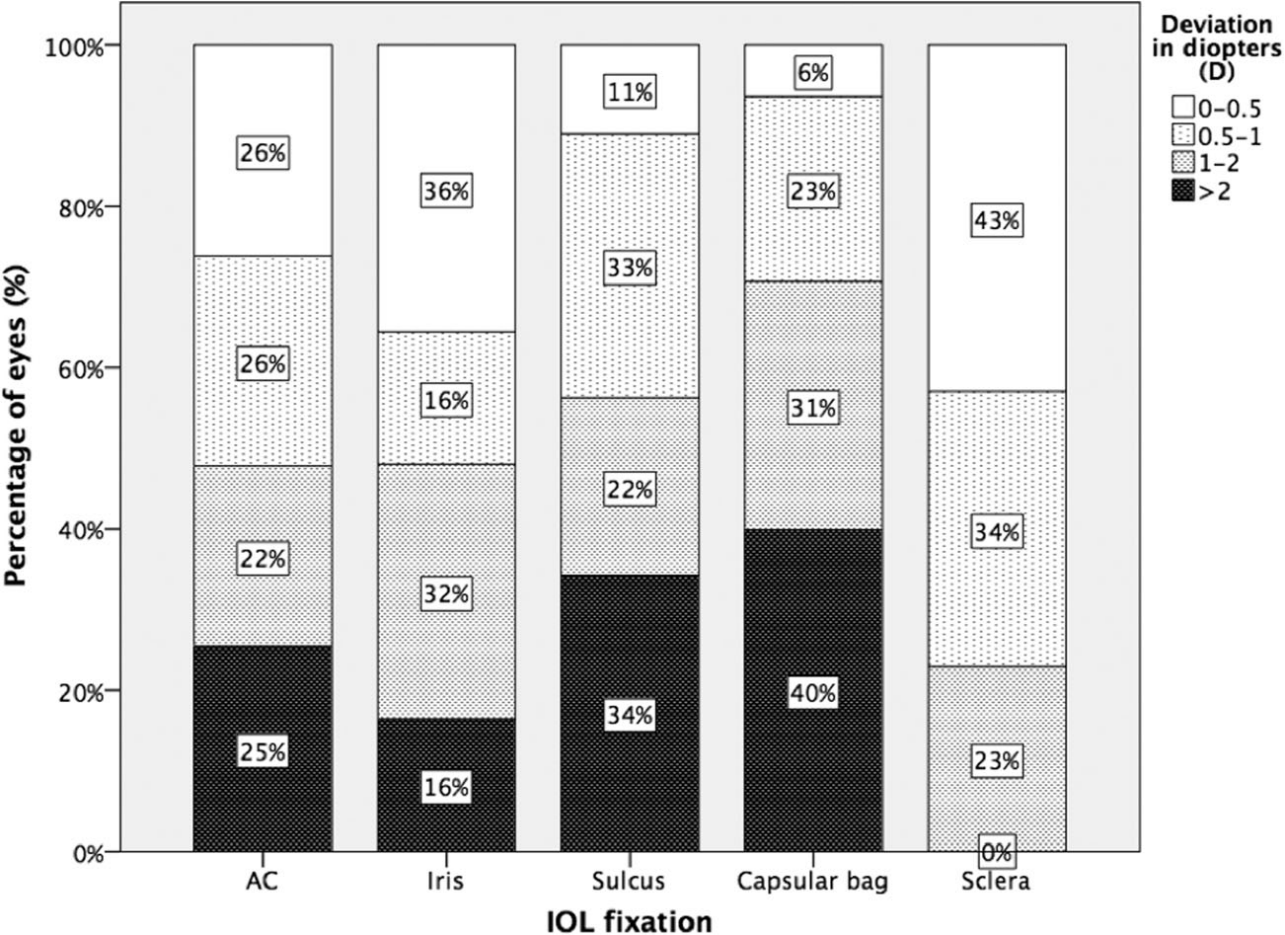
Percentage of eyes with a deviation of absolute error (AE) between 0 and 0.5D, 0.5 and 1D, 1 and 2D, and more than 2D

The percentages of the eyes with a deviation of AE from 0 to 0.5D, 0.5 to 1D, 1 to 2D, and over 2D with regard to IOL fixation are demonstrated in Fig. 3. Scleral fixation had the highest percentage of eyes with an AE between 0 and 1D, which corresponds to a refractive PE within ± 1D, but the low number of evaluated eyes of this group (6) does not allow reliable further conclusions. Almost 50% of the eyes that received IC-IOLs or AC-IOLs had a refractive PE within ± 1D, showing equally good results. However, 36% of IC-IOLs showed a refractive PE within ± 0.5D compared to 26% of AC-IOLs, demonstrating a slight superiority of IC-IOLs.

**Fig. 4:**
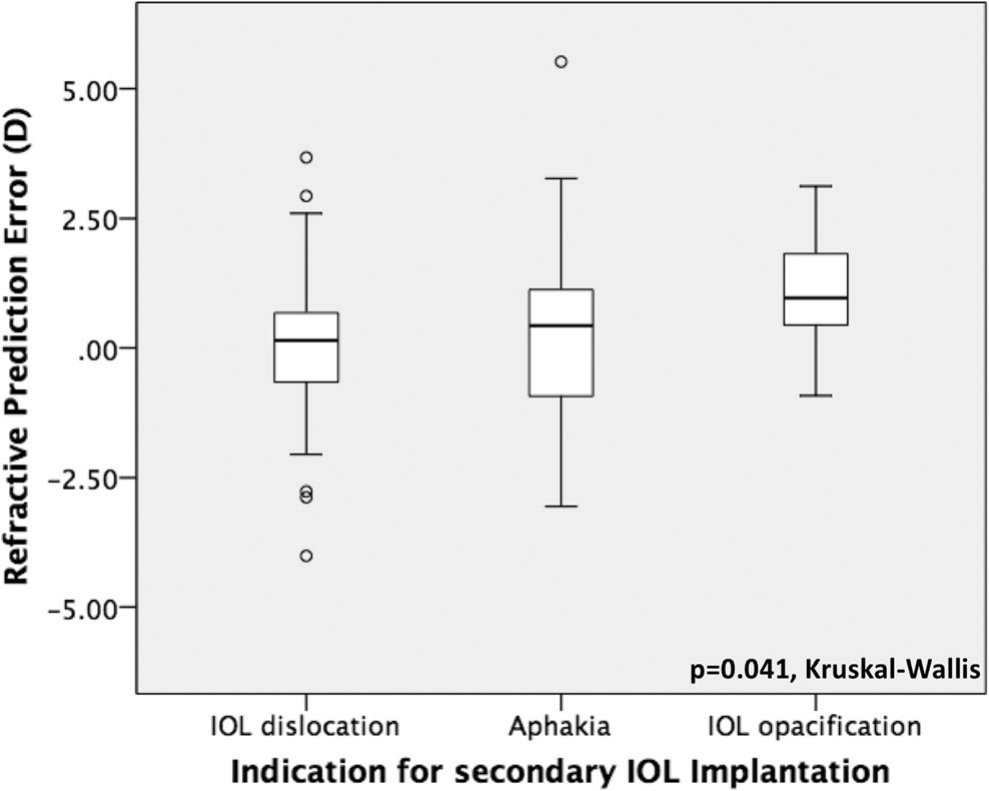
Boxplot analysis of refractive prediction error in diopters (D) with regard to the indication for secondary intraocular lens implantation

**Fig. 5:**
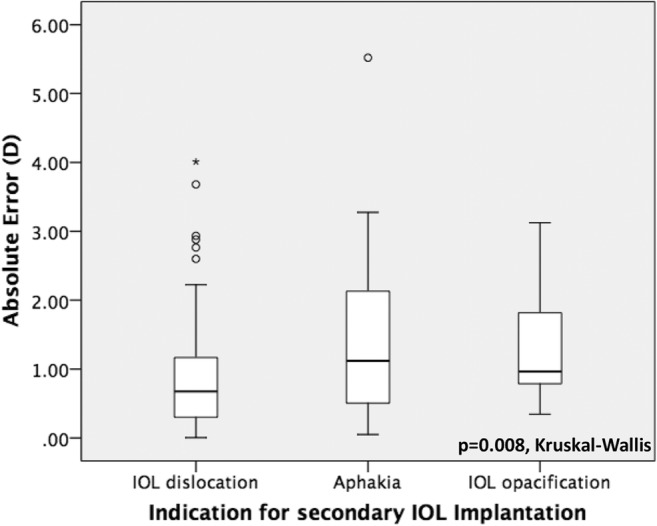
Boxplot analysis of mean absolute error in diopters (D) with regard to the indication for secondary intraocular lens implantation

**Fig. 6:**
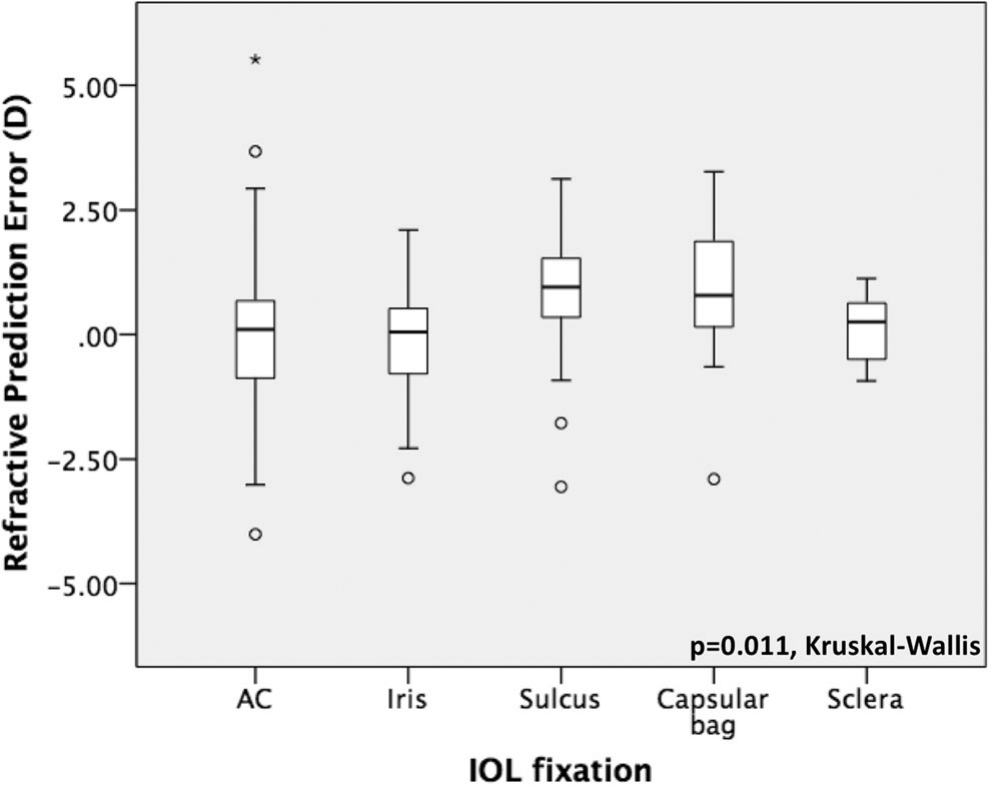
Boxplot analysis of refractive prediction error in diopters (D) with regard to the type of the secondary intraocular lens fixation. AC anterior chamber, IC iris-claw

**Fig. 7:**
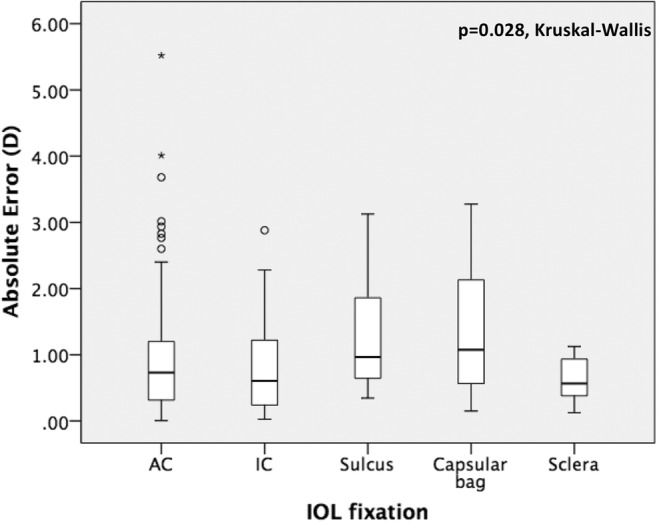
Boxplot analysis of absolute error in diopters (D) with regard to the type of the secondary intraocular lens fixation. AC anterior chamber, IC iris-claw

Prediction and absolute error were statistically significantly different with regard to the indication for secondary IOL surgery (*p* = 0.041 and *p* = 0.008, respectively, Kruskall-Wallis) and the type of fixation of the IOL (*p* = 0.011 and *p* = 0.028, respectively, Kruskall-Wallis) as demonstrated in Figs. 4, 5, 6, and 7. No further correlations were found between refractive prediction error or absolute error and any preoperative, intraoperative, and postoperative factors.

Additionally, regarding the AE, no difference was detected between the different biometric formulas that were used or the surgeon (*p* = 0.393, and *p* = 0.306, respectively, Kruskall-Wallis). Moreover, no correlation was detected between AE and axial length (*r* = 0.057, *p* = 0.467, Pearson correlation).

AE did not differ significantly whether or not an AC hemorrhage occurred (*p* = 0.13, Mann-Whitney *U* test). Furthermore, no differences of AE were observed with regard to all other complications. In general, eyes without postoperative complications demonstrated a lower AE, although not to a statistically significant degree.

### Postoperative complications

Defined short-term postoperative complications were mainly anterior chamber hemorrhage (14%) and bulbus hypotony (6%). Other short-term complications included choroidal detachment (1×), IOL dislocation (2×), IOL decentration (1×), high intraocular pressure (3×), bullous keratopathy (1×), prolonged intraocular inflammation (2×), and iris capture (3×). Three months after surgery, complications reported included cystoid macular edema (2×), IOL dislocation (6×), IOL decentration (3×), and IOL opacifications in one case at a later time.

### Postoperative anterior chamber hemorrhage

Postoperative AC hemorrhage was not affected by any preoperative risk factor or by the indication for surgery (*p* = 0.309, chi-square test). On the other hand, the fixation’s position of the IOL was significantly associated with the rate of postoperative AC hemorrhage. In particular, a significantly higher rate of AC hemorrhage was observed after retropupillary iris-claw fixation, whereas all other fixation positions showed lower bleeding rates (*p* = 0.003, chi-square test).

### Postoperative hypotony

Postoperative hypotony was defined as IOP < 6 mmHg within 2 days after surgery. In contrast, persisting hypotony was defined as decreased IOP at 6 weeks’ follow-up. Eyes with uveitis in their record showed a significantly higher rate of postoperative hypotony after surgery (*p* = 0.026, chi-square test), but no other risk factors showed any influence on postoperative hypotony.

Indication for secondary IOL implantation, type of IOL fixation or surgeon did not affect the postoperative hypotony rate (*p* = 0.664, *p* = 0.609, and *p* = 0.364, respectively, chi-square test).

### Other complications

At 3 months, the overall rate of complications was highly dependent on the type of secondary IOL fixation (*p* = 0.015, chi-square test). Specifically, sclera fixated IOLs showed a high complication rate after 3 months with two new IOL dislocations out of six eyes, whereas IC-IOLs and AC-IOLs showed a low complication rate.

## Discussion

While epidemiologic data referring to the incidence of IOL dislocation shows an incidence of 0.1–3% of implanted IOL after 5 years, most experts agree that the general incidence of secondary IOL implantation due to different reasons is increasing [2, 4].

IOL dislocation, aphakia (from various causes), IOL opacifications, patient dissatisfaction after multifocal intraocular lens implantation, or UGH syndrome constitute the major reasons for surgery [5]. This study was conducted in order to examine the incidence of preoperative ocular risk factors and indications for surgery in a large European vitreoretinal referral center, and to compare the complications rate and the refractive and visual outcome.

Different parameters were examined for their incidence as preoperative risk factors. While PXF is known to be a main risk factor for zonular instability and IOL dislocation with a presence of over 30% [5–7], recent data reveals that PXF is frequently clinically underdiagnosed in cases of late in-the-bag IOL dislocation [8, 9]. Although in our study IOL dislocation was the major reason for surgery, PXF was present only in 12% of all cases, in 21 eyes with an IOL dislocation (16%) and specifically in 18 of 112 eyes with an in-the-bag IOL dislocation (16%). Regarding the low incidence of clinically detected PXF in our cases of dislocated IOL as compared to other studies, we cannot rule out that the incidence of PXF may have been underreported.

Further factors like trauma, previous retinal surgery, vitrectomy, uveitis, myopia, and aging are also reported in the literature [2, 10–13] as risk factors. Interestingly, in this study, previous complete pars plana vitrectomy was the most frequent preoperative ocular risk factor for in-the-bag IOL dislocation compared to other studies [10, 14]. With increasing rates of vitreoretinal surgery, this might also reflect a factor in increasing incidence of IOL dislocation overall. On the other hand, this study was conducted in a referral center for vitreoretinal surgery, so these data might be influenced by the specific patient population.

The population’s average age in this cohort was almost a decade younger than is reported in most studies [9, 15]. This might reflect a specific patient population in this series. On the other hand, the incidence of previous trauma or uveitis was higher than in other published data [2, 5, 12, 16]. In our series, about 25% had either a previous trauma or a history of uveitis, representing a relative high incidence of these risk factors in comparison to other studies [5, 16]. The high incidence of previous vitrectomy, previous retinal surgery, previous trauma, uveitis, and the younger age reflect the complexity of the cases of this cohort, and the need for sufficient refractive results. Intraocular lens dislocation has been noted as the major reason for secondary IOL implantations [16]. In this study, the IOL dislocation was also the leading cause for surgery, with the majority of the eyes having an in-the-bag IOL dislocation. Further reasons were secondary aphakia or IOL opacifications. Eleven aphakic eyes had a previous trauma, whereas the remaining 25 eyes (69%) were aphakic due to other causes. Although the spectrum for surgery indication is quite wide, IOL dislocation and aphakia after complicated cataract surgery or other complicated intraocular surgery still remain the major reasons for secondary IOL implantation in most other recent studies as well [5, 16]. The mean time of 8.7 years between secondary IOL implantation and primary cataract surgery found in our study is in accordance with published data [5, 17].

The postoperative objective refractive spherical equivalent (ORSE) showed no statistically significant difference between the groups with regard to the fixation position of the secondary IOL (*p* = 0.106, Kruskal-Wallis). In contrast, refractive prediction error as well as absolute error differ statistically significantly between the different IOL positions. Postoperative ORSE and AE of this study are comparable to the published data [18, 19]. In our study, the retropupillary IC-IOLs (Verisyse®) showed the lowest AE, followed by the AC-IOLs, sulcus IOLs, and PC-IOLs, although the difference was not statistically significant. Scleral fixated IOLs were excluded from the statistical analysis due to re-fixation of the same IOL in 6 of the 12 eyes and small number of the remaining cases, but they showed comparable results to IC-IOLs, as already published in other studies [20]. In contrast to Brunin et al., sulcus fixated IOLs showed a relatively high PE and AE [16]. The fact that PE and AE show a higher deviation might be due to the fact that on an empirical basis, IOL Power was weakened upon implantation in sulcus. Thus, the intended refraction as predicted in biometry would not correspond to the targeted refraction as there is an empiric corrective factor. This assumption is supported by the fact that the use of biometric formulas did not show different results for the AE in these cases. Previous retinal surgery or trauma was noted as a risk factor in 6 of the 10 eyes with a capsular bag secondary IOL implantation, whereas a vitrectomy was performed in combination with the secondary in-the-bag IOL implantation in 8 of these 10 cases. We think that this context as well as the complexity of the cases might explain the relative high AE that was observed. Additionally, a relative high AE may have occurred due to the complexity of the preoperative optical biometry, especially in cases of IOL dislocation, where the capsular-bag/lens complex may interfere with the optical axis and lead to a further error in calculating the optimum refractive power of the IOL to be implanted.

Although preoperative CDVA in our study was worse than in other published literature, an improvement of CDVA was observed in all groups, except of sclera fixated IOLs [16]. Interestingly, this group showed in our study the best pre- and postoperative CDVA and no change after surgery, but the small number of patients of this group and the variable results do not allow any further conclusions. Considering the complexity of the cases of this study and the existing preoperative visual impairment, mainly due to previous vitrectomy or retinal surgery, only 50.9% of the eyes had a preoperative CDVA better than 0.5 LogMAR. However, 79.4% achieved a postoperative CDVA better than 0.5 LogMAR, whereas 60% of the eyes showed an improvement of CDVA.

The frequency of the postoperative AC hemorrhage in our study was not higher than other published data and was most frequently observed after iris fixation, followed by scleral fixation. AC hemorrhage did not affect the final CDVA and postoperative AE negatively. As already reported, a small self-limiting AC hemorrhage is not likely to worsen the postoperative visual acuity [2, 21].

Additionally, in this study, a low rate of postoperative hypotony was observed, compared to Rey et al. and Dajee et al. [19, 21]. A reason for that could be a smaller incision procedure or the low incidence of uveitis cases. Despite the complexity of the cases, cystoid macular edema, elevated intraocular pressure, and IOL dislocation incidence were comparable to the published literature [5, 16, 22, 23]. However, IOL dislocation occurred only in two IC-IOLs and in one AC-IOL, indicating a relative low incidence. In contrast to that, two of the 12 scleral fixated IOLs showed a dislocation and one a decentration, reflecting a rather higher complication rate. However, all these eyes underwent simultaneously complete pars plana vitrectomy, which may have affected their postoperative stability.

Predictability of the magnitude of postoperative refraction, which is expressed by AE, showed in our study correlation with indication for surgery, fixation type, and postoperative AC hemorrhage. Moreover, this study reports a lower AE in cases with dislocated IOLs compared to aphakia or IOL opacification. The highest refractive deviations were documented for the correction of secondary aphakia. In 8 of these cases, an IOL was implanted in-the-bag, whereas in 8 other cases in sulcus. However, in these groups, a large proportion of eyes have had previous trauma or vitrectomy for various reasons. Additionally, the empiric correction of the sulcus position was not reflected in the AE.

Regarding the defined as long-term complications, IOL decentration and dislocation were the most frequently observed. Interestingly, these occurred more often in eyes with secondary IOL implantation in the sulcus or in-the-bag and are not commonly observed in AC-IOLs or IC-IOLs or sclera fixated IOLs in our study. Thus, it seems that in our study with a large portion of eyes having suffered trauma, secondary IOL as AC-IOL or IC-IOL or scleral fixation is not only safer in the long term but also shows a better predictable refractive outcome with the limitations mentioned above.

Our study has several shortcomings. Firstly, it is a retrospective study with all its limitations. With regard to this retrospective design, we believe that our results rather underreport the results as patients with unsatisfying results may tend to ask for revisions. We assume that the big number of patients that preferred to undergo further follow up examinations at their ophthalmologist rather than at the clinic were satisfied with their refractive outcome. However, the lack of follow up limits the population of this cohort and most probably leads to reporting rather the results of the most complex cases, which intend to visit big referral centers for their follow up. Secondly, several surgeons using different techniques and skills were involved, which does not correspond to a standardized procedure. Additionally, this study is not limited to one condition, but also includes complicated cases such as, for example, traumatic cases.

We cannot comment on the outcome of a specific technique from our data as the surgical procedure was chosen according to the surgeons’ experience. In our setting, scleral fixation was chosen if other options were not feasible. In general, these were complex or traumatic cases. Thus, our results concerning the scleral fixation technique rather underreport the results of this technique [16]. Additionally, this technique has been modified recently and the current technique only partly corresponds to the evaluated time period [24].

Our study analyzes a large number of cases with the need for secondary IOL implantation. While surgical standards in these rather complex cases are difficult to investigate, we believe that all of these cases represent indications for secondary IOL implantation and thus are worth reporting. While one may argue in individual cases which is the preferable technique, all of these techniques have their particular indication. This claim is underlined by our results showing no significant differences in most of these techniques with regard to visual acuity, PE, and AE.

As a conclusion, we may state that our study shows that in our setting, vitrectomy seems to be a major preoperative ocular risk factor in all eyes that underwent secondary IOL implantation surgery. Additionally, all applied techniques show satisfying refractive and visual results albeit not being comparable to primary IOL implantation in the capsular bag. Retropupillary IC-IOLs showed the best refractive results, though not statistically significantly different to the other fixation positions. However, we claim that IC-IOLs seem to be the most satisfying option of secondary IOL implantation especially in complicated cases, with history of previous vitrectomy and should be preferred. Further studies analyzing techniques with recent biometric formulas and techniques in a standardized setting are warranted.
